# Upregulation of Neuroinflammatory Protein Biomarkers in Acute Rhegmatogenous Retinal Detachments

**DOI:** 10.3390/life13010118

**Published:** 2022-12-31

**Authors:** Minali Prasad, Jia Xu, Joshua S. Agranat, Weiming Xia, Sarah Daley, Steven Ness, Xuejing Chen, Nicole H. Siegel, Thor D. Stein, Jaeyoon Chung, Manju L. Subramanian

**Affiliations:** 1 Department of Ophthalmology, Boston University School of Medicine & Boston Medical Center, Boston, MA 02118, USA; 2 Department of Pharmacology and Experimental Therapeutics, Boston University School of Medicine, 72 East Concord Street, Boston, MA 02118, USA; 3 Geriatric Research Education and Clinical Center, VA Bedford Healthcare System, Bedford, MA 01730, USA; 4 Laboratory Medicine, Department of Pathology, Boston University School of Medicine, Boston, MA 02118, USA; 5 VA Bedford Healthcare System, Bedford, MA 01730, USA; 6 VA Boston Healthcare System, Boston, MA 02130, USA; 7 Department of Medicine (Biomedical Genetics), Boston University School of Medicine, 72 East Concord Street, Boston, MA 02118, USA

**Keywords:** rhegmatogenous retinal detachment, neuroinflammatory markers, fibroblast growth factor, monocyte chemoattractant protein-1, inducible protein-10, macrophage inflammatory protein-1 beta, interleukin 8, interleukin 6

## Abstract

The purpose of this study is to characterize the inflammatory cytokine profile in rhegmatogenous retinal detachments (RRDs) compared to surgical controls. Vitreous humor was collected from patients undergoing vitrectomy for RRD and noninflammatory vitreoretinal diseases. A quantitative immunoassay was used to measure the levels of 36 cytokine markers. Linear regression analysis with the duration of detachment as the predictor and log-transformed cytokine levels as the outcome was conducted for normally distributed cytokines as determined by the Shapiro–Wilk test. The analysis was adjusted for age, sex, and race. The Kruskal–Wallis test was used for cytokines not normally distributed. Twenty-seven RRD cases and thirteen control cases were studied. Between all RRDs and controls, fibroblast growth factor 2 (FGF2) (*p* = 0.0029), inducible protein-10(IP-10) (*p* = 0.0021), monocyte chemoattractant protein-1 (MCP-1) (*p* = 0.0040), interleukin (IL)-16 (*p* = 0.018), IL-8 (*p* = 0.0148), IL-6 (*p* = 0.0071), eotaxin (*p* = 0.0323), macrophage inflammatory protein (MIP)-1 alpha (*p* = 0.0149), MIP-1 beta (*p* = 0.0032), and the thymus and activation regulated cytokine (TARC) (*p* = 0.0121) were elevated in RRD cases. Between acute RRDs (n = 16) and controls, FGF2 (*p* = 0.0001), IP10 (*p* = 0.0027), MCP-1 (*p* = 0.0015), MIP-1β (*p* = 0.0004), IL-8 (*p* = 0.0146), and IL-6 (*p* = 0.0031) were elevated. Determining alterations in inflammatory cytokine profiles may aid in understanding their impact on RRD development, clinical course, and complications before and after surgical repair.

## 1. Introduction

Rhegmatogenous retinal detachment (RRD) is a vision-threatening condition in which mechanical forces at the vitreoretinal interface lead to a break in the retina, allowing for the passage of fluid into the subretinal space, separating the retina from the retinal pigment epithelium (RPE) and choroid [1]. This condition affects 1 in 10,000 people per year and occurs more frequently in males [2,3]. RRD is the most common type of retinal detachment, and patients can present with symptoms including flashes of light, visual floaters, and loss of vision [2]. Risk factors for RRD include lattice degeneration, myopia, prior cataract surgery, prior retinal detachment, and trauma [3,4,5]. In adults, RRDs are typically an isolated eye condition and are not associated with any systemic diseases, in contrast to other types of retinal detachments such as tractional and serous retinal detachments, which can occur from advanced diabetic retinopathy and inflammatory eye conditions, respectively. In pediatric patients, over half of RRDs are associated with Stickler Syndrome, a systemic collagenopathy [6].

The goal of treatment of RRDs is to reattach the retina to the RPE with surgical techniques such as pneumatic cryopexy, scleral buckle, or pars plana vitrectomy [7]. In general, single-surgery success rates are high in these procedures, but each approach has its own indications and complications profile. After surgical repair, some patients have minimal to no visual improvement despite successful anatomic reattachment of the retina. There are numerous pre-operative predictive factors for the outcomes of RRDs. Studies have shown that worse pre-operative visual acuity (VA), macula-off status, longer duration of detachment, the presence of proliferative vitreoretinopathy (PVR), older age, male sex, and non-White race portend poorer visual outcomes after treatment [3,8,9,10,11]. The etiology of poor visual outcomes is thought to be due to photoreceptor death from increased intraocular inflammation caused by prolonged detachment, especially within the macula or recurrent detachments requiring multiple repairs that can damage photoreceptors beyond recovery after reattachment [12,13,14]. Inflammation contributes to the formation of PVR, which is the most common cause of failure after RRD repair and leads to an increased risk of recurrent RRD. PVR is characterized by the development of contractile fibrocellular epiretinal or subretinal membranes and, at times, intrinsic intraretinal fibrosis [15,16,17]. The exact inflammatory response that contributes to the development of PVR after RRD repair is not fully understood.

Previous studies have found elevated levels of inflammatory cytokines in patients with RRD; however, the reported cytokine profiles are variable, and the lack of consistency in the data warrants further study (Table A1 and Table A2) [18,19,20,21,22,23,24,25,26,27,28,29,30,31,32,33]. 

The aim of this study is to further characterize the nature of inflammation in patients with RRD by evaluating their vitreous cytokine profile compared to controls, with results stratified based on the duration of detachment.

## 2. Materials and Methods

- Inclusion Criteria

This study was approved by the Institutional Review Board of Boston Medical Center (BMC) and Boston University Medical Campus Institutional Review Board and adheres to the tenets of the Declaration of Helsinki. Patients enrolled in this prospective, cross-sectional study were 18 years or older with a primary language of English or Spanish and scheduled for a pars plana vitrectomy in at least one eye. For patients included in this study, the following demographic variables were collected from their medical charts: age, sex, and race. Self-reported racial categories included White, Black or African American, American Indian or Alaska Native, Asian, and Native Hawaiian or Other Pacific Islander according to the U.S. Census Bureau guidelines [34]. The study group included patients with RRD, and the control group included patients with non-inflammatory eye conditions including visually significant floaters, vitreomacular traction (VMT), macular hole (MH), epiretinal membrane (ERM), and subluxed crystalline lens with an intact capsule and no eye inflammation. All patients in both groups underwent vitrectomy alone with the exception of one combined case that included phacoemulsification, and in that patient, the vitreous specimen was retrieved prior to the phacoemulsification. Subjects enrolled in this study were part of a larger cohort of 95 participants that included patients requiring surgery for several indications, and cases that did not include a diagnosis of RRD were not included in this study [35]. 

Cases were grouped based on the duration of detachment, either less than 2 weeks or greater than 2 weeks, defined as the onset of symptoms such as flashes, floaters, decreased vision, and peripheral visual field loss. Symptoms were used as a proxy for the duration of detachment since this was the only way to clinically estimate RRD duration. We stratified the RRD cases at 2 weeks because it is at this point that a prior study defined RRD as chronic: [36] (1) Less than or equal to two weeks (hereafter described as “acute RRD”) and (2) greater than two weeks (hereafter described as “chronic RRD”).

b. Biospecimen Collection and Analysis

Prior to starting the infusion during pars plana vitrectomy, 0.5–1.0 mL of undiluted vitreous fluid was aspirated by the vitrector into an attached 3 mL syringe [37,38,39,40]. Using a sterile technique, the syringe with the patient’s vitreous fluid was capped and labeled with a study number. The samples were stored on ice during transportation, centrifuged for 5 min at 12,000 rpm, and aliquoted and stored at −80 C until analysis. At the time of analysis, 200 µL samples were prepared with 100 µL of vitreous fluid diluted 1:1 with 1% Blocker A (MSD #R3BA 4) in wash buffer. The Meso Scale Discovery MULTI-SPOT Assay System Neuroinflammation Panel 1 was used to complete a quantitative immunoassay for 36 neuroinflammatory factors. Duplicate samples were quantified, signal detection was conducted using a sulfo-tag conjugated secondary antibody, and analyte levels (pg/mL) were measured with an MSD SECTOR S 600 Imager. The samples were analyzed for the following proteins: Fibroblast growth factor 2 (FGF2), C-reactive protein (CRP), Eotaxin, Eotaxin-3, Fms-like tyrosine kinase-1 (Flt-1), intercellular adhesion molecule 1 (ICAM1), interferon-gamma (IFN-γ), interleukin (IL)-10, IL-12p70, IL-13, IL-15, IL-16, IL-17A, IL-1α, IL-1β, IL-2, IL-4, IL-5, IL-6, IL-7, IL-8, interferon-gamma inducible protein-1 (IP10), monocyte chemoattractant protein-1 (MCP-1), macrophage inflammatory protein-1 alpha (MIP1α), MIP1β, serum amyloid A protein (SAA), thymus and activation regulated chemokine (TARC), Tie-2, tumor necrosis factor-alpha (TNFα), TNF-β, vascular cell adhesion molecule 1 (VCAM-1), VEGF, VEGF-C, and VEGF-D. The Neuroinflammation Panel 1 kit was obtained from MSD (catalog number K15210D-1, Meso Scale Discovery, Rockville, MD, USA).

c. Statistical Analysis

Statistical analysis was conducted using SAS v 9.4. The Shapiro–Wilk test was used to determine the normality of log-transformed cytokine levels. If the cytokine levels were normally distributed, we used a linear regression model controlling for age, race, and sex to compare mean cytokine levels between groups. We did not adjust for lens status since it has been found that lens status does not significantly impact cytokine levels [19]. If the cytokine levels were not normally distributed, the nonparametric Kruskal–Wallis test was used to compare mean cytokine levels between groups. Concentrations of vitreous cytokines were log-transformed after adding 1 to achieve a normal distribution [41,42,43], given that the linear regression analysis requires normal distribution of the outcome variable (cytokine levels). Log transformation is commonly used in biomedical research [44,45,46,47] and allows the use of a normal distribution to model continuous outcomes in skewed data. Since the classic bell-shaped normal distribution does not always describe observed data in real life, log transformation converts the skewed data into a more normally distributed dataset compared to the data prior to log transformation. As a result, parametric tests such as linear regression models can be used to analyze the data. 

In the primary analysis, we compared mean cytokine levels between RRD cases and controls. In the secondary analysis, we compared mean cytokine levels between acute RRD cases (≤2 weeks duration) and controls. We did not complete a subgroup analysis comparing chronic RRD cases against controls or acute cases due to large variation in duration (3 weeks to 8 months), smaller sample size, and greater likelihood of recall bias by patients as the length of time from symptom onset and the chronicity of the retinal detachment increased. To correct for multiple comparisons, the p-values of cytokines were adjusted to account for potential type 1 errors using the false discovery rate (FDR), and we focused on the cytokines that were statistically significant (*p* ≤ 0.05) and within an FDR of 10%. We provided effect size and standard error for normally distributed cytokines analyzed with a linear regression model. We were not able to report effect sizes for cytokines analyzed with the Kruskal–Wallis test due to the fact that the SAS does not report effect sizes for this nonparametric test.

Fold changes represent ratios of mean log-transformed cytokine levels and were calculated by dividing mean log-transformed cytokine levels between cases and controls [48]. The following ratios (using log-transformed cytokine concentrations) were used to calculate fold changes: Acute RRD/controls and all RRD cases/controls.

## 3. Results

Of the 95 subjects enrolled in the study, 80 samples were collected. Fifteen subjects’ samples were dropped due to the following reasons: Inability to obtain the sample or insufficient sample collection (n = 5), loss to follow-up (n = 3), surgery cancellation (n = 4), mislabeled specimens (n = 2), and withdrawal of consent (n = 1). An additional forty subjects (out of eighty) were excluded as their surgical indication was not RRD or did not meet the criteria for the control group. In total, 40 patients were included in this study, comprising 27 subjects with RRD (11 subjects with chronic RRD and 16 with acute RRD) and 13 controls without RRD (Table 1). 

Demographic information of our subjects is listed in Table 2. The mean age of the controls is higher than that of the cases, but it was not statistically significant (*p* = 0.0926). White patients comprised just under 50% of cases and controls. RRD cases and controls were similar with respect to gender distribution (*p* = 0.1862). Four of the eleven chronic cases had preoperative PVR. 

Table 3 shows the mean cytokine levels before and after log transformation. The results of the primary and secondary analysis are shown in Table 4. Of the ten cytokines that significantly increased from controls to all RRD cases, six cytokines were found to be significantly upregulated in those with acute RRD cases. Fold changes from Table 4 were calculated using mean log-transformed cytokine levels from Table 3.

## 4. Discussion

This study found that certain inflammatory markers were significantly increased in RRDs, including FGF2, IP10, MCP-1, IL-16, MIP-1α, MIP-1β, IL-8, Eotaxin, TARC, and IL-6. These findings are consistent with previously published studies (Table A2). Additionally, a subset of the above cytokines was elevated specifically in the acute stage of RRDs, including FGF2, IP10, MCP-1, MIP-1β, IL-8, and IL-6, and this has not been previously reported. 

Some of the cytokines analyzed in this study have been implicated in the inflammatory causes of photoreceptor death. MCP-1, released by Muller cells, induces resident microglia migration and subsequent microglia activation and secretion of cytotoxic factors [49]. Activated microglia and dead photoreceptors promote a further increase in MCP-1 levels in a pro-inflammatory positive feedback loop [50]. The presence of other cytokines from this study, including IL-1β, IL-6, IL-7, and TNF-α, was found to increase MCP-1 levels, further contributing to photoreceptor death [12]. Of the aforementioned cytokines, MCP-1 is also upregulated in the acute phase of RRD. By measuring cytokine levels at various time points after detachment, it may be possible to determine which cytokines are involved early in the feedback loop and guide further studies in preventing photoreceptor death. 

As previously mentioned, inflammation contributes to the formation of PVR, the most common cause of surgical treatment failure. While this study identifies a subset of cytokines upregulated within the first two weeks of detachment, correlating cytokine levels within this subset with those involved in the later development of PVR may provide insight into the biochemical pathways associated with PVR. Understanding the cytokines that trigger the cascade of fibrosis in some postoperative eyes but not others will help further work in preventing PVR in order to achieve better surgical and visual outcomes.

Among the interleukins tested, IL-8 and IL-6 were found to be upregulated between RRD cases and controls. IL-6 is known to stimulate B-cells, hematopoiesis, and the production of acute-phase proteins [51,52]. IL-6 receptor blockers reduced subretinal fibrosis and prevented PVR by reactivating the platelet-derived growth factor. IL-8 is produced by phagocytes and mesenchymal cells and activates, recruits, and promotes extravasation and the respiratory burst of neutrophils. Several studies suggest that chemoattraction and neutrophils are involved in the retinal detachment and PVR disease processes [20,22]. Furthermore, Takahashi et al. hypothesized that IL-8 levels could reflect the level of photoreceptor damage given that IL-8 was found to be positively correlated with the extent of detachment, and photoreceptor cell damage indirectly increases IL-8 expression [23]. 

Non-interleukin growth factor cytokines significantly upregulated in acute RRD includes FGF2. FGF2 stimulates endothelial cells, promotes angiogenesis and wound healing, and leads to the proliferation of Müller cells, retinal astrocytes, and retinal pigment epithelial cells [53,54]. Multiple studies have demonstrated that FGF2 levels are elevated in patients with PVR but not in patients with primary RDs without PVR (Table A2) [26,27,55,56]. In this study, FGF2 was elevated in acute RRD, but no cases developed PVR after surgical repair, possibly because the timing of surgical intervention in the acute stage prevented its development. Future studies may consider stratifying FGF2 levels by the duration of detachment and FGF2 levels after surgical repair in PVR to further study the involvement of FGF2 in RRD development and progression. 

IP10, MCP-1, and MIP-1β are non-interleukin cytokines involved in monocyte chemotaxis and activation and were upregulated in this study. Yang et al. found that MCP-1 can activate monocytes that induce RPE apoptosis and increase levels of intracellular calcium and reactive oxygen species [57]. MCP-1 may lead to lead to photoreceptor death and poor visual outcomes after successful anatomic repair of RRDs. Similarly, MIP-1β promotes the migration and adhesion of macrophages and microglia [58,59]. Additionally, IP-10 is a pro-inflammatory chemoattractant for monocytes and macrophages and functions as an anti-angiogenic and antifibrotic agent (Table A2) [20,21,22]. Therefore, while IP-10 may attract leukocytes to the inflamed area of retinal detachment, it may also counteract the fibrotic actions of MCP-1 and MIP-1β as proposed by Takahashi et. al. [23]. 

The strength of this study lies in isolating acute RRD cases by the duration of detachment and analyzing cytokine profiles while adjusting for demographic variables, particularly patient race. Because most previous studies use nonparametric tests that cannot adjust for demographic covariates, adjusting for demographic variables, such as race and sex, has rarely been performed in most prior studies (Table A2) [18,22,23,24,30]. Furthermore, by applying an a priori false discovery rate of 10% to correct for multiple comparisons, we were conservative in our approach by focusing on cytokines that met this threshold. Our study also had limitations. The most significant limitation is our sample size of 27 RRD cases and 13 controls; however, our sample sizes are comparable to prior studies of vitreous cytokine profiles in RRD (Table A2) [18,20,21,22,23,24,25,26,28,32]. There is a 3:1 ratio of males to females among RRD cases due to the low sample size, and because more males than females consented to the original study [40]. Subgroup analysis of chronic RRD (greater than 2 weeks duration) was not performed in this study because we were limited by the heterogeneity of the chronicity (range of duration of detachment: 3 weeks to 8 months) and small sample size (n = 11). Additionally, patient-reported durations of detachment for chronic cases were very likely subject to greater recall bias as the length of time from symptom onset and the chronicity of the retinal detachment increased. Another potential limitation is the use of symptoms as a marker of the duration of detachment, which relies on subjective patient reporting. However, in clinical practice, symptom duration is the only proxy available for approximating the duration of RRD and informs decision making for the type of surgical intervention and timing of surgery. Lastly, the error in the cytokine level exceeds the mean cytokine level in some cases (Table 3). However, log transformation addressed this issue by converting the original dataset into one that is more normally distributed. Future studies may be able to investigate the utility of the vitreous cytokine profile in RRD to ascertain the duration of detachment without relying on the patient-reported onset of symptoms. 

In conclusion, we corroborated the findings of elevated cytokines in RRD and identified a subset of inflammatory markers that may be early markers of RRD. Our findings may be foundational for future studies aiming to elucidate the effects of these cytokines on visual and anatomic outcomes after surgical repair, understand the pathogenesis of long-term consequences, such as PVR, and identify potential targets for the prevention of those complications as well as therapeutic interventions.

## Figures and Tables

**Table 1:** Duration of detachment among RRD patients and surgical indications among control patients.

RRD CASES	N = 27 (5 Female)	%
Acute (≤2 weeks)	16 (3 Female)	59
Chronic (>2 weeks)	11 (2 Female)	41
**CONTROLS**	**N = 13 (6 Female)**	**%**
Visually Significant Non-inflammatory Floaters	2	15.4
Vitreomacular Traction	3	23.1
Macular hole	6	46.2
Secondary Epiretinal Membrane	1	7.7
Subluxed Crystalline Lens	1	7.7

**Table 2:** Demographics of Study Subjects Comparing RRD Cases and Controls.

	RRD Cases				Controls	
	Chronic (n = 11)	%	Acute (n = 16)	%	Controls (n = 13)	%
**Sex**						
Male	9	81.82	13	81.25	7	53.85
Female	2	18.18	3	18.75	6	46.15
**Mean Age**	46.45		55.06		61.08	
**Race**	2	18.18	11	68.75		
White	5	45.45	2	12.50	6	46.15
Black	1	9.09	2	12.50	4	30.77
Asian American	2	18.18	1	6.25	0	0
Indian/Alaskan Native	1	9.09	0	0	1	7.69
Unknown	9	81.82	13	81.25	2	15.38

**Table 3:** Mean and standard deviation (SD) of cytokine levels (pg/mL) before and after log-transformation.

	Mean ± SD Cytokine Level (pg/mL)			Mean ± SD Log-Transformed Cytokine Level		
Cytokine	Control	Acute	Chronic	Control	Acute	Chronic
FGF2	1.42 ± 1.81	79.42 ± 134.46	890.27 ± 1525.29	0.96 ± 0.95	4.46 ± 2.57	5.77 ± 4.64
IP10	3278.13 ± 7126.34	4196.03 ± 4336.28	12546.28 ± 24553.54	12.24 ± 3.50	9.28 ± 1.14	11.55 ± 1.90
MCP-1	3502.39 ± 4596.02	7219.01 ± 4855.979	11710.61 ± 13705.72	10.28 ± 3.25	12.60 ± 0.77	12.56 ± 1.76
IL-16	10.40 ± 9.19	19.94 ± 27.02	35.81 ± 30.90	3.14 ± 1.08	3.88 ± 1.07	4.50 ± 1.71
MIP-1α	24.51 ± 26.91	38.56 ± 28.60	84.55 ± 127.57	3.79 ± 1.82	4.81 ± 1.46	5.66 ± 1.33
MIP-1β	35.28 ± 34.49	78.04 ± 45.99	83.29 ± 88.20	4.67 ± 1.29	6.06 ± 0.89	5.78 ± 1.46
IL-8	14.61 ± 12.68	48.09 ± 67.65	73.56 ± 130.14	12.24 ± 1.66	4.97 ± 1.29	4.79 ± 2.14
Eotaxin	96.03 ± 151.51	89.06 ± 59.43	109.14 ± 57.61	5.68 ± 1.72	5.89 ± 1.80	6.57 ± 0.87
TARC	11.18 ± 26.61	39.20 ± 85.77	24.02 ± 41.05	2.14 ± 1.87	3.62 ± 2.22	3.56 ± 1.74
IL-6	17.70 ± 44.54	80.49 ± 239.44	546.09 ± 1229.72	2.17 ± 2.05	3.97 ± 2.28	4.05 ± 3.93

**Table 4:** Regression/Kruskal–Wallis Analysis Results for Vitreous Cytokines in RRD. All significant cytokines in both the primary and secondary analysis with *p*-value < 0.05 and FDR < 0.1 are bolded. * denotes cytokines analyzed with the linear regression model.

	Primary Analysis (All RRD vs. Controls)				Secondary Analysis (Acute RRD vs. Controls)			
Cytokine	Effect Size (SE)	*p*-Value	FDR	Fold Change (RRD/Control)	Effect Size (SE)	*p*-Value	FDR	Fold Change (Acute RRD/Control)
FGF2 *	**3.6657 (1.1377)**	**0.0029**	**0.03**	**5.20**	**3.9852 (0.8544)**	**0.0001**	**0.0030**	**4.64**
IP10 *	**2.7305 (0.8144)**	**0.0021**	**0.03**	**1.24**	**3.1553 (0.9291)**	**0.0027**	**0.0186**	**1.27**
MCP-1 *	**2.4562 (0.7921)**	**0.004**	**0.03**	**1.22**	**3.1302 (0.9233)**	**0.0015**	**0.015**	**1.23**
IL-16	-	0.018	0.06	1.31	-	0.0357	0.153	1.24
MIP-1α	-	0.0149	0.0559	1.36	-	0.0535	0.2006	1.27
MIP-1β *	**1.3898 (0.4361)**	**0.0032**	**0.03**	**1.27**	**1.8077 (0.4308)**	**0.0004**	**0.006**	**1.30**
IL-8 *	**1.5854 (0.6154)**	**0.0148**	**0.0559**	**1.48**	**1.6971 (0.6375)**	**0.0146**	**0.073**	**1.50**
Eotaxin	-	0.0323	0.0969	1.09	-	0.1225	0.2127	1.04
TARC	-	0.0121	0.0559	1.68	-	0.0959	0.2127	1.70
**IL-6**	**-**	**0.0071**	**0.0426**	**1.84**	**-**	**0.0031**	**0.0186**	**1.83**

## Data Availability

The data presented in this study are available upon request from the corresponding author. The data are not publicly available due to their containing information that could compromise the privacy of research participants.

## Appendix A

**Table A1:** Abbreviations and the corresponding full form of the biomarkers examined in the present work or in the literature cited in Table A2.

Abbreviation	Full Form
BDNF	Brain-derived neurotrophic factor
CCL1	C-C motif chemokine ligand 1
CCL2	C-C motif chemokine ligand 2
CCL3	C-C motif chemokine ligand 3
CCL4	C-C motif chemokine ligand 4
CCL7	C-C motif chemokine ligand 7
CCL11	C-C motif chemokine ligand 11
CCL13	C-C motif chemokine ligand 13
CCL19	C-C motif chemokine ligand 19
CCL26	C-C motif chemokine ligand 26
CRP	C-reactive protein
CTACK	Cutaneous T cell-attracting chemokine
CXCL9 (MIG)	C-X-C motif chemokine ligand 9 (Monocyte induced by gamma)
CXCL10	C-X-C motif chemokine ligand 10
CXCL11	C-X-C motif chemokine ligand 11
CXLC5	C-X-C motif chemokine ligand 5
G-CSF	Granulocyte Colony Stimulating Factor
GM-CSF	Granulocyte-macrophage colony stimulating factor
GRO	Human growth-regulated oncogene
FGF-2	Fibroblast growth factor-2
Flt-1	Fms-like tyrosine kinase-1
ICAM-1	Intracellular adhesion molecule-1
IFN-α2	Interferon-alpha-2
IFN-γ	Interferon gamma
IL-1α	Interleukin-1 alpha
IL-1β	Interleukin 1-beta
IL-1RA	Interleukin-1 receptor antagonist
IL-2	Interleukin-2
IL-4	Interleukin-4
IL-5	Interleukin-5
IL-6	Interleukin-6
IL-7	Interleukin-7
IL-8	Interleukin-8
IL-10	Interleukin-10
IL-11	Interleukin-11
IL-12	Interleukin-12
IL12-p40	Interleukin-12 (p40)
IL-13	Interleukin-13
IL-15	Interleukin-15
IL-16	Interleukin-16
IL-17A	Interleukin-17A
IL-18	Interleukin-18
IP-10	Interferon gamma-induced protein 10
MCP-1	Monocyte Chemoattractant Protein-1
MCP-3	Monocyte Chemoattractant Protein-3
MDC	Macrophage-derived chemokine
MIF	Macrophage migration inhibitory factor
MIP-1α	Macrophage inflammatory protein-1 alpha
MIP-1β	Macrophage inflammatory protein-1 beta
PDGF	Platelet derived growth factor
PDGF-AA	Platelet derived growth factor AA
PDGF-AB	Platelet derived growth factor AB
PDGF-AB	Platelet derived growth factor BB
PEDF	Pigment Epithelium-Derived Factor
SAA	Serum amyloid A
sCD137	Soluble tumor necrosis factor receptor superfamily member 9
SDF-1α	Stromal cell-derived factor-1 alpha
sFAS	Soluble Fas cell surface death receptor
TARC	Thymus and activation regulated chemokine
TGF-α	Transforming growth factor alpha
TGF-β1	Transforming growth factor beta 1
TGF-β2	Transforming growth factor beta 2
TGF-β3	Transforming growth factor beta 3
Tie2	Endothelial specific receptor tyrosine kinase 2
TIMP-1	TIMP metallopeptidase inhibitor 1
TIMP-2	TIMP metallopeptidase inhibitor 2
TNF-α	Tumor necrosis factor alpha
TNF-β	Tumor necrosis factor beta
VCAM-1	Vascular adhesion molecule 1
VEGF	Vascular endothelial growth factor
VEGFA	Vascular endothelial growth factor A
VEGFC	Vascular endothelial growth factor C
VEGFD	Vascular endothelial growth factor D

**Table A2:** Abridged compilation of vitreous humor cytokine studies comparing RRD cases and controls.

Study	Methodology	Controls	Statistical Tests	Findings
Danielescu et. al. [18]	RRD (n = 40) vs. Controls (n = 20)	Epiretinal membrane (ERM), macular hole (MH)	-	↑ in RRD: G-CSF ↑ in PVR: MCP-1
Garweg et. al. [19]	RRD (n = 71) vs. Controls (n = 26)	MH	Mann–Whitney U-test, Kruskal–Wallis H test	↑ in RRD with ≥10-fold upregulation: CXLC5, CCL26, CCL1, IL-6, CXCL11, CCL7, CCL13, MIG/CXCL9, CCL19 and TGF-β1
Kiang et. al. [20]	RRD (n = 24) vs. Controls (n = 10)	Vitreous opacities, ERM	Regression adjusted for age, sex, duration and extent of detachment with additional nonparametric tests	↑ in RRD: eotaxin, fractalkine, GRO, IFN-α2, IFNγ, IP-10, MCP-1, MCP-3, MDC, MIP-1α, MIP-1β, IL-1RA, IL-6, IL-7, IL-8, IL12-p40, FGF-2, G-CSF, GM-CSF, PDGF-AA, PDGF-AB/BB, TGF-α, VEGFA, sCD137, sFAS
Kunikata et. al. [21]	RRD (n = 19) vs. Controls (n = 17)	ERM, MH	Mann–Whitney U test	↑ in RRD: IL-6, IFNγ, MCP-1, MIP-1β, eotaxin, IP-10, IL-8, VEGF, G-CSF
Balogh et. al. [22]	RD (RRD without PVR (n = 30), PVR (n = 16), and PDR with traction retinal detachment (TRD) (n = 8)) vs. Controls (n = 19)	ERM	Kruskal–Wallis analysis of variance	↑ in RRD, PVR, and PDR: IL-6, IL-16, IFNγ, MCP-1, MIF ↑ in PVR and PDR: IL-8, eotaxin, IP-10, SDF-1α ↑ in PDR only: CTACK, VEGF, IL-18
Takahashi et. al. [23]	1. RRD (n = 28) vs. MH (n = 14) 2. RRD vs. Proliferative Diabetic Retinopathy (n = 55)	MH (negative control)PDR (positive control)	Kruskal–Wallis ANOVA, post-hoc Mann–Whitney U test	↑ in RRD compared to MH: IL-6, IL-8, MCP-1, MIP-1β, IP-10, ↑ in RRD compared to ↑ in RRD compared to PDR: IL-6, IL-8, IL-10, IL-12, IL-13, PDGF, VEGF
Rasier et. al. [24]	RRD (n = 22) vs. Controls (n = 12)	ERM, MH	Student’s t test and Mann–Whitney U test	↑ in RRD: VEGF, IL-8
Conart et. al. [25]	RRD (n = 41) vs. Controls (n = 33)	MH, vitreomacular traction	-	↑ in RRD: IL-1RA, IL-6, IL-7, IL-8, IFN-γ, CCL2, CCL3, CCL4, CXCL10 and CCL11, G-CSF
La Heij et. al. [26]	PVR (n = 53) vs. Controls (n = 20)	MH, macular pucker	-	↑ in PVR: IL-6, FGF2
Kon et. al. [27]	1. Patients with RRD and preoperative PVR vs. those without PVR 2. Patients with RRD and postoperative PVR vs. those without PVR	N/A	-	↑ in RRD and preoperative RRD: TGF-β2, FGF2, IL-1β ↑ in RRD and postoperative RRD: TGF-β2, IL-6, FGF2
Capeans et. al. [28]	RRD (n = 43) vs. Controls (n = 18)	-	-	↑ in PVR: MCP-1
Mitamura et. al. [29]	1. PVR (n = 74) vs. RRD (n = 22) 2. PVR vs. Controls (n = 26)	ERM, MH	-	↑ in PVR compared to RRD and controls: MIF
Yoshimura et. al. [30]	RRD (n = 63) vs. Controls (n = 83)	ERM, MH	Mann–Whitney U test and Kruskal–Wallis test	↑ in RRD: IL-6, IL-8, MCP-1
Pollreisz et. al. [31]	RRD (n = 60) vs. Controls (n = 20)	ERM	Single t-test	↑ in RRD: TIMP-1, TIMP-2, MIP-1α, MCP-1, IL-6, IL-8, IP-10, BDNF, TGF-β3, PDGF-AA, PDGF-BB
Ogata et. al. [32]	1. RRD (n = 26) vs. Controls (n = 14) 2. PVR (n = 6) vs. Controls	MH	-	RRD: ↑ PEDF PVR: ↓ PEDF, ↑ VEGF
Limb et. al. [33]	RRD (n = 35) vs. Controls (n = 22)	MH	Mann–Whitney U test	↑ in RRD: ICAM-1

“-“ denotes unknown information. “↑” and “↓” denote upregulated and downregulated biomarkers in the experimental group (ie RRD cases) compared to controls, respectively.
